# Effect of Surface Structure and Adsorption Activity on Implanting of b-Oriented ZSM-5 Zeolite Film on Modified α-Quartz Substrate

**DOI:** 10.3389/fchem.2019.00636

**Published:** 2019-09-18

**Authors:** Ruizhi Chu, Deguang Yang, Xianliang Meng, Shi Yu, Yongzhou Wan, Jiaxing Wu, Jian Wang

**Affiliations:** ^1^Key Laboratory of Coal Processing and Efficient Utilization of Ministry of Ministry of Education, Xuzhou, China; ^2^School of Chemical Engineering and Technology, China University of Mining and Technology, Xuzhou, China

**Keywords:** b-oriented ZSM-5 zeolite film, α-quartz substrate, modification, surface structure, adsorption activity

## Abstract

b-oriented ZSM-5 zeolite film was synthesized on the macropore α-quartz substrate modified with titanium dioxide (TiO_2_), polyvinyl acetate (PVA), and chitosan (CTS) by hydrothermal crystallization. By comparing the binding energy and b-oriented angle of zeolite film on each modified α-quartz substrate, the orientations, and combinations derived from structure-adsorption relationship were investigated with Material Studio simulation. Furthermore, the effects of calcination temperature and ultraviolet (UV) irradiation time on the surface structure and adsorption activity of TiO_2_ coating were studied. The increase adsorption potential energy and the formation of Ti-O-Si bind between zeolite crystal phase and substrate facilitate the continuous and uniform zeolite film growth. The TiO_2_ interlayer with anatase phase after UV irradiation presents a smooth surface with high Ti-OH density, consequently to high selectivity of b-orientation growth for the ZSM-5 crystals. Compared with the traditional ZSM-5, the higher stability has been exhibited with b-oriented ZSM-5 film /TiO_2_/α-quartz in the MTA reaction, and the methanol conversion and BTX selectivity remained higher than 90 and 70%, after 6 h reaction.

**Graphical Abstract:**
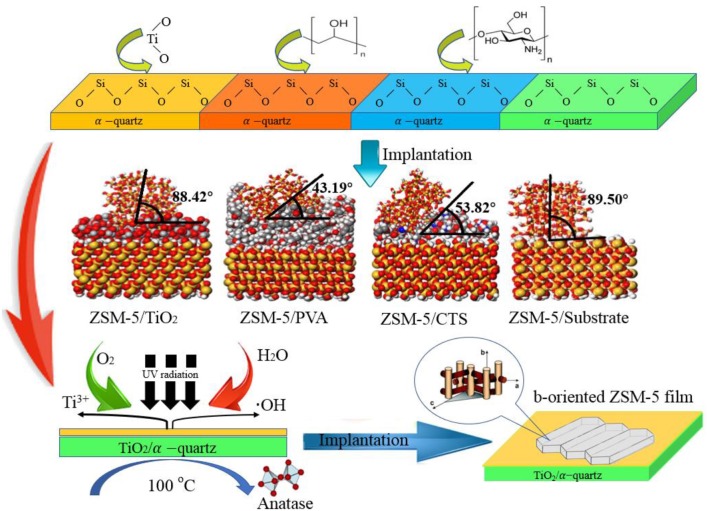
Surface modification mechanism of α-quartz substrate.

## Highlights

- The b-oriented ZSM-5 zeolite film was prepared on the substrate of modified α-quartz.
- The modification-structure-adsorption relationship of modifiers and α-quartz surface were investigated.
- The orientations and combinations derived from structures and adsorption between modified α-quartz and ZSM-5-crystallites were studied.

## Introduction

Aromatic hydrocarbons, especially light aromatic hydrocarbons such as benzene, toluene, and xylene (BTX) are important raw materials for organic chemicals and polymer industry (Ye et al., 2019). The production of light aromatic hydrocarbons BTX by methanol conversion (MTA) is of great significance to realize the low-cost synthesis of value-added chemicals via non-oil routes and to expand the application of methanol (Ali et al., 2015; Bozzano and Manenti, 2016; Mitsuyoshi et al., 2017).

At present, some MTA studies are reported and the main reaction catalysts are ZSM-5 (Na et al., 2018), ZSM-22 (Catizzone et al., 2017), MCM-22 (Lacarriere et al., 2011), MCM-36 (Lacarriere et al., 2011), and H-Beta molecular sieve (Svelle et al., 1999) etc. ZSM-5 exhibits excellent shape-selectivity catalysis for BTX aromatic hydrocarbons due to the suitable diameter of the tunnel, therefore it becomes the most widely studied catalyst for MTA reaction (Li et al., 2003; Khodakov et al., 2010). However, aromatic hydrocarbon selectivity and carbon deposition on ZSM-5 remain as the core issues which make it difficult to scale-up for MTA industry deployment.

Significant efforts have been attempted to address the carbon deposition issue on catalysts during MTA reaction, for example, some studies have proposed to synthesize nanometer acicular c-axis ZSM-5 catalyst (Shen et al., 2013). The length of the main reaction channel of a, b orientation is in favor of strengthen the diffusion process, thereby suppresses carbon deposition. For example, Shen et al. (2013, 2014). Used acid-treatment to slowly dissolve the aluminum and silicon from natural dolomite, and formed the Al–Si–O structural unit in the catalyst synthesis. Without directing agent, the Al-Si-O structural unit was self-assembled and crystallized into a ZSM-5 acicular nanometer powder along c-axis, The MTA reactivity of this acicular ZSM-5 nanoparticles was evaluated and it is found that the reaction molecules were able to rapidly diffuse through the micropores in the short axes of a and b, thus the diffusion limit is lowered. Compared with the traditional non-b-oriented ZSM-5, the residence time of the reaction molecules in the pores of this novel materials is shortened, therefore the carbon deposition on the catalyst is significantly reduced, which contributes to the prolonged catalyst life and improved BTX selectivity. However, the study also found that the crystals of acicular molecular sieves are so brittle, which limits their industrial application due to the difficulties for storage and transportation.

To solve above-mentioned issues, our group proposed to utilize the adsorption and diffusion advantages of ZSM-5 in the b-oriented straight channel (Lai et al., 2010; Zhou et al., 2013). The b-oriented ZSM-5 one-dimensional molecular sieve was loaded on the nanospheres with mesoporous pore structure to construct the b-oriented ZSM-5 nanosphere shell material with micro-mesoporous pore structure. The micropores of the material will become the active center of the MTA catalyst with excellent carbon deposition resistance and BTX selectivity, meanwhile the mesopores work as fast channels for the diffusion of aromatic BTX small molecules.

It was found that the pore structure and surface activity of the substrate exhibit significant effects on film orientation and adhesion strength (Wei and Yen, 2007). Therefore, to improve the binding force between the crystal nucleus and the substrate surface, some attempts are made to increase the surface activity and adsorption strength of the substrate by employing suitable coupling agents or modifiers. For example, Lee et al. (2000b) used 3-Aminopropyltriethoxysilane and 3-Glycidoxypropyltrimethoxysilane as coupling agents to synthesize the ZSM-5 with a great b-oriented crystal layer on the glass surface. In addition, this repeatable synthesis of the molecular sieve onto the substrate was demonstrated by similar mechanism (Lee et al., 2000a, 2001; Park et al., 2002; Jin et al., 2006). Yeung et al. (Jlh et al., 2000) assembled randomly oriented silicalite-1 molecular sieves on the surface of stainless steel. Fu et al. (2018) prepared a silicalite-1 molecular sieve onto the ceramic surface by manual assembly method, and obtained dense seed layer of b-oriented crystals. However, due to the lack of fundamental understanding, the research is still mainly in the sample screening stage with different substrates and modifiers via trail-and-error approach. Different from other chemical processes, the surface of the colloidal microspheres is not destroyed during the loading of the crystal nucleus on the surface of the substrate. The nanocrystal nucleus firstly adsorbs on the colloidal microsphere surface, whose structure and properties can then affect the formation process of ZSM-5 crystal nuclei. Therefore, the surface structure and properties of colloidal microspheres were introduced and investigated in the molecular design and synthesis system of microspheres with core-shell structure. The effects of the surface structure of colloidal microspheres, as well as the surface properties of nanocrystal cores and modifier molecules were studied. To achieve the fundamental understanding of the directed growth of molecular sieve crystals, the effect of structural on the b-oriented adsorption of nanocrystal nuclei on the surface of colloidal microspheres was investigated. It is of important significance for both the theoretical and practical design of novel core-shell molecular sieve.

In addition, another goal of synthesis the b-oriented ZSM-5 zeolite is to increase the bonding force of the crystal nucleus on the substrate surface. The substrate materials with limited pore channels are reported in most present studies, which greatly restricts the application of molecular sieve film in separation and catalysis. Therefore, the design strategy to obtain monodisperse oriented molecular sieve film on the multi-channel substrate surface has become a challenging and important research topic in the field of oriented film materials.

Furthermore, as for the preparation of b-oriented ZSM-5 zeolite film on the surface of macropore substrate, the influencing factors of the oriented growth and assembly of nanocrystalline core are regarded as the essential points for the preparation process. In this paper, a series of ZSM-5 zeolite films anchoring on the α-quartz substrate surface will be synthesized by hydrothermal crystallization. Both organic (polyvinyl acetate and chitosan) and inorganic (titanium dioxide) modifiers are applied to coat on the prepared samples. Considering the macropore α-quartz substrate, the effects of substrate surface structure and adsorption activity on the b-oriented adsorption of nanocrystal nuclei will be investigated.

## Experimental Section

### Simulation of Substrate Surface Modification and ZSM-5 Zeolite Combination Process

**Figure 1:**
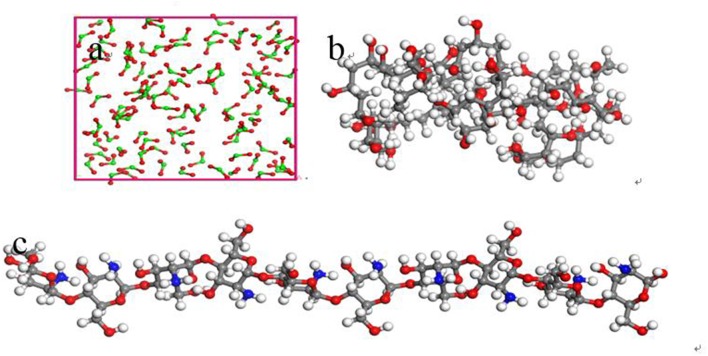
Schematic diagram of modifier model **(a)** Inorganic modifier TiO_2_, **(b)** grid structure organic modifier PVA, and **(c)** linear organic modifier CTS (Gray, white, red, and green represent C, H, O, and Ti, respectively).

The inorganic modifier titanium dioxide (TiO_2_) and organic modifier polyvinyl acetate (PVA) and chitosan (CTS) were modeled by 3Dviewer in Material Studio software. In order to make the simulation conditions close to the molecular weight and polymerization degree of the organic modifier in the experiment, the polymerization degrees of PVA and CTS were set to 40 and 10 (Razmimanesh et al., 2015; Wei et al., 2017), respectively. The repeating unit of the TiO_2_ molecule was set to 100 (Baguer et al., 2009). The model is shown in Figure 1. Molecular dynamics simulation of the adsorption process of ZSM-5(010) crystal plane on the modified surface was performed under NVT system at 450 K, with a step size of 1 fs and simulation time of 1,000 ps using the Universal force field of Forcite module.

### Substrate Surface Modification Experiment

The surface of the substrate was modified by inorganic modifier (TiO_2_) and organic modifier (PVA and CTS). TiO_2_ (10wt.%, Hang Zhou Wan Jing new materials co., LTD.) precursor oxides sols were coated on the substrate surface by sol-gel method, and dried for 12 h at 50°C, then roasted for 2 h at 100^o^C. Chitosan (0.5 wt.%, Sinopharm Chemical Reagent Co., Ltd.) and PVA (4Wt.%, Sinopharm Chemical Reagent Co., Ltd.) were coated on the surface of the substrate for three times. It was dried at 30°C and 60% relative humidity for 12 h subsequently dried at 60°C for 2 h.

In order to study the effect of Ti-OH species and its concentration on the substrate surface of the supported ZSM-5 crystal layer, the samples substrate loaded with TiO_2_ coating was calcined in muffle furnace at 100, 300, 600, and 900^o^C, irradiated with UV for 2, 4, 6, and 8 h, respectively.

### Preparation of b-Oriented ZSM-5 Zeolite Film

The ZSM-5 zeolite film was supported on the α-quartz substrate by hydrothermal synthesis. A prescribed amount of Tetrapropylammonium Hydroxide (TPAOH) (10 wt.%, Sinopharm Chemical Reagent Co., Ltd.) was added to the DI water followed by dropwise adding Ethyl Orthosilicate (TEOS) (Xiyu Chemical Co., Ltd.) and aluminum nitrate. Subsequently the mixture was aged for 24 h to obtain a clear and uniform solution. The synthesis mole composition was 0.32TPAOH: 1SiO_2_: 0.005Al_2_O_3_: 165H_2_O. The substrate was vertically inserted into a hydrothermal crystallization vessel with a Teflon liner, and the synthesis solution was added to the crystallization vessel without passing through the substrate. After heating to 165^o^C for 80 min, the obtained catalyst then is slowly rinsed for several times with DI water. Finally, the catalysts are calcinated at 550°C for 2 h in a tubular furnace with a heating rate of 0.5°C/min.

### Characterizations

The microstructure of the modified substrate and the surface of the ZSM-5 film was characterized by scanning electron microscopy. Prior to the test, the sample was subjected to gold spray treatment. XRD data was obtained on a VERTEX 80v X-ray diffractometer using Cu Kα radiation (*k* = 0.15418 nm), scanning speed 2°/min, scanning range 5^−^50°, tube current and tube voltage of 150 mA, 40 kV, respectively. The contact angle was measured by the JC2000C4 contact angle meter and measured by a synthetic liquid solid drop method. Each contact angle was an average value of multiple measurements at different positions on the surface at room temperature. Nitrogen (N_2_) adsorption/desorption isotherms were measured with a physical adsorption instrument (ASAP2010) at −196°C. The textural properties of α-quartz substrate was determined by the high-performance automatic mercury intrusion meter (AutoPore IV 9510). The total pore volume (V_total_) was derived from the amount of N_2_ adsorbed at p/p_0_ = 0.99, the BET method was applied to determine the total surface area (S_total_), the t-plot method is specifically used to identify micropores, and the BJH method was used to estimate the size distribution.

### The Catalytic Activity and Selectivity of ZSM-5 Zeolite in MTA Reaction

The catalytic activity and selectivity in MTA reaction were studied in a fixed-bed reactor. 0.5 g of catalyst was carried out in a reaction tube with an inner diameter of 10 mm, the catalyst was activated for 2 h at 450°C, and the methanol was introduced at a space velocity (WHSV) of 2.85 h^−1^ under normal pressure at 470°C, the concentration of the components was analyzed using a gas chromatograph.

## Results and Discussion

### Effect of Modifier Species on Preparation of b-Oriented ZSM-5 Zeolite Film

#### Characterization of ZSM-5 Zeolite Film: Composition, Morphology

**Figure 2:**
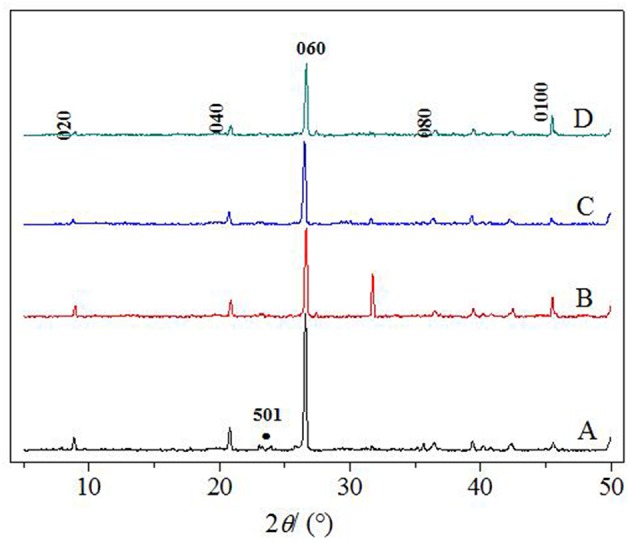
XRD patterns of ZSM-5 zeolite film on **(A)** uncoated, **(B)** TiO_2_, **(C)** CTS, and **(D)** PVA coating.

In this paper, TiO_2_, PVA, and CTS coatings were used to modify the surface of macropore α-quartz substrate, and the addition of intermediate oxide layer, to change the directional growth of ZSM-5 zeolite film on the substrate. Figure 2 shows the XRD patterns of ZSM-5 zeolite film with various coatings. There are five diffraction peaks at 2θ = 8.89°, 17.81°, 26.82°, 36.06°, and 45.46°, attributed to the (020), (040), (060), (080), and (0100) crystal faces of MFI zeolite. The results indicate that ZSM-5 zeolite film with b-oriented crystals perpendicular to the substrate surface was formed. Compared with the other three modifiers, Figure 2B (0h0) shows the strongest diffraction peak intensity, implying the TiO_2_ is more favorable for the growth of b-oriented ZSM-5 zeolite film. In addition, the uncoated substrate exhibits a weak characteristic peak at 2θ = 23.3° (Figure 2A), attributed to the (501) crystal plane which was identified as ZSM-5 in the a-axis (Chu et al., 2016), indicating the presence of the small amount of a-axis crystals.

**Figure 3:**
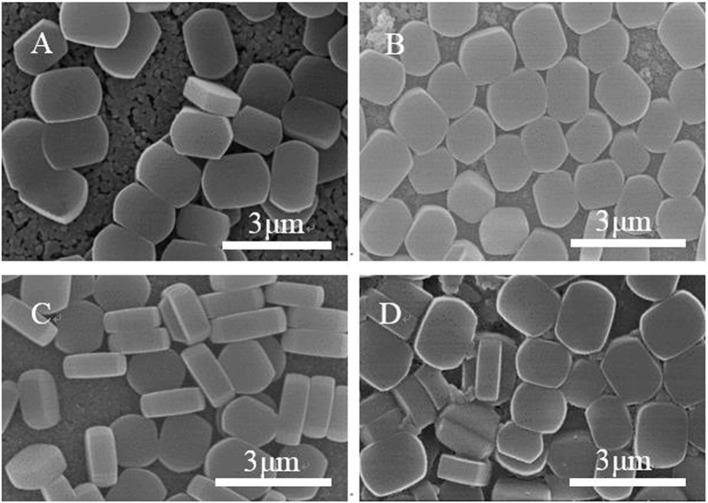
SEM images of ZSM-5 zeolite film on **(A)** uncoated, **(B)** TiO_2_, **(C)** CTS, and **(D)** PVA coating.

Figure 3 shows SEM images of ZSM-5 zeolite film synthesized on the surface of substrates with different modifiers. Most of the ZSM-5 zeolite crystals grow in the b-orientation with coffin shape, and the effective TiO_2_ modification is clearly observed. Compared with the unmodified matrix (Figure 3A), others show that the ZSM-5 crystals increase significantly. This indicates that in the presence of the intermediate modification layer, the ZSM-5 molecular sieve nucleus can be more easily anchored and grown on the substrate, exhibiting higher coverage, and better film formation property. Moreover, the crystals on the substrate coated with organic modifiers (CTS and PVA) exhibit greater superposition and non-b-orientation than that with TiO_2_. Part of the ZSM-5 crystals are embedded in the intermediate modifier layer during growth, with the uneven crystals distribution and low coverage. Besides, the degree of orientation is inconsistent due to the difference in surface structure and adsorption activity between the surface of the ZSM-5 zeolite film and the substrate.

#### Substrate Surface Structure Analysis

**Figure 4:**
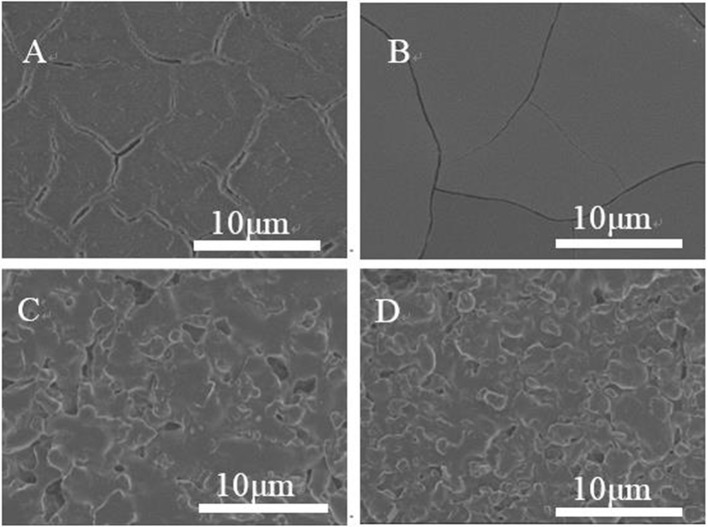
SEM diagram of substrate coated with different modifiers **(A)** uncoated, **(B)** TiO2, **(C)** CTS, and **(D)** PVA.

It is found that the microstructure of the substrate is one of the important factors affecting the orientation growth of the crystal on the substrate. Figure 4 shows the SEM morphology of the substrates to analyze their microstructures with different coatings. The inorganic oxides in the nanosized particles can be effectively coated on flat surface (Figure 4B), in contrast the organic modifier film in a mesh or linear is tightly attached to form an uneven surface (Figures 4C,D), This observation indicates that the smooth nanocrystalline core-substrate interface promotes the growth of ZSM-5 zeolite film., Di et al. modified the surface of the smooth glass substrate by sol-gel method to obtain different surface coverages of silicalite-1 film (Di et al., 2011). Previous studies have shown that -OH-rich substrate with smooth surface does not own the effective adsorption through hydrogen bonding, and this may be cause by the structural differences between the surfaces of the material (Fu et al., 2018). We characterized a series of substrate materials (dispersed in deionized H_2_O) using ζ-potential measurements. It is found that both SiO_2_ and silicalite-1 exhibit negative surface charges, and the mixture of these two materials shows a −34.6 mV ζ-potential value, which lies between the values of their individual components, ζ-potential value of TiO_2_ is close to zero, revealing the weak interactions on adsorption. This electrostatic adsorption potential of smooth inorganic TiO_2_ surface results in the growth of ZSM-5 zeolite film. For organic modifiers, the film surface is positively charged and covered by large number of -OH and -NH_2_ active groups. In the meantime, the organic modifier is dehydrated during the coating process, the colloidal particles are aggregated due to the intermolecular force and chemical force, resulting in the uneven distribution of the surface active group on the matrix and different physical adsorptions. Thereby it further explains the degree of accumulation and aggregation of ZSM-5 crystals on the organic modifier surface.

#### Adsorption Activity

There is significant difference of the adsorption force (van der Waals force) between the modifier interface and the zeolite crystal phase (silica particles), this adsorption force can be characterized by non-zero frequency Hamaker constant A_v>0_ to evaluate the ability of various substrates to initiate surface gelation. According to the literature (Ji et al., 2012), the Hamaker constant A_v>0_ of SiO_2_-SiO_2_ and SiO_2_-TiO_2_ interaction are 0.35 × 10^−20^ and 3.1 × 10^−20^, respectively. The values suggest the silica–silica adsorption force is much weaker than silica–titanium adsorption force, which further explains that the poorer quality of the ZSM-5 film on substrate than that on TiO_2_ layer. According to the expressions and constants of the literature (Lai et al., 2000), we estimate the Hamaker constant A_v>0_ of SiO_2_-PVC and SiO_2_-CTS interaction are 1.82 × 10^−20^ and 1.53 × 10^−20^, respectively. By comparison, it is proved that the adsorption force of the substrate surface coated with TiO_2_ is obviously larger than that of the other modifiers. Thus, it can be concluded that the substrate with strong adsorption force is favorable for the loading of the crystals.

**Figure 5:**
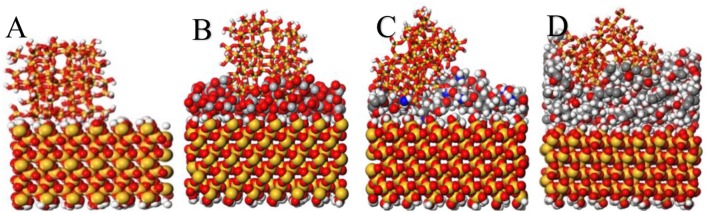
Adsorption simulation of ZSM-5 nanometer crystal (010) on the surface of modifier **(A)** substrate, **(B)** TiO_2_, **(C)** CTS, and **(D)** PVA.

ZSM-5 was pre-planted in the b-direction on the surface of TiO_2_, CTS, and PVA coated substrates, the orientation angle are 43.19, 53.82, and 88.42° (Table 1), respectively, indicating that the orientation angles of ZSM-5 grown on the modifier in the b-orientation decreases in the order: TiO_2_ > CTS > PVA. The CTS and PVA are rich in -OH groups and -NH_2_ groups, which can generate hydrogen bonds at the interface between ZSM-5 and the substrate, thereby generating interaction forces. However, with the help of kinetic simulation in Figures 5C,D, it is found that although there is a strong interaction between macromolecules of CTS/PVA and the substrates, a small range of cross-linking or agglomeration will occur on the surface of the substrates. Therefore, the exposed surfaces of the CTS and PVA modifiers are always accompanied by a slight roughness, resulting in a certain inclination angle. As shown in Figure 5A, when the ZSM-5 coated with TiO_2_ modification on the substrate for the plant, ZSM-5 embedded in the modified layer with b-orientation. As small molecule, TiO_2_ does not agglomerate on the surface of the substrate, meanwhile a strong interaction force can be maintained between TiO_2_ molecules, thereby a dense and uniform modification layer can be formed on the substrate. Further, stable Ti-O-Si bond was formed due to the interaction between Ti-OH with Si-OH, which facilitated the ZSM-5 embedded in the TiO_2_ modified layer. However, the Si-O-Si bond was not formed at the uncoated substrate effectively, and the van der Waals force becomes the main adsorption force in the interfaces, resulting in failure to form an effective pre-planted (Figure 5B). Consequently, the b-oriented ZSM-5 is best pre-planted on the substrate coated by TiO_2_.

**Table 1:** Adsorption characteristics of ZSM-5 nanocrystalline crystal (010) surface and modifier surface/substrate.

	**Substrate**	**TiO_**2**_**	**CTS**	**PVA**
b-oriented Angle (°)	89.5	88.42	53.82	43.19
Binding energy (Kcal/mol)	−218.64	−752.17	−335.00	−458.35

Meanwhile, even though the TiO_2_ modifier can provide the great b-oriented angle and maximum interaction energy, the Figure 3B shows that the ZSM-5 zeolite film still has a small amount of non-b-orientation ones, which may be caused by the differences in the species and density of -OH groups on the substrate surface. Moreover, the layer of TiO_2_ was modified to study the effect of Ti-OH species and their density on the crystal orientation as well as surface coverage of ZSM-5 zeolite film.

### Effect of TiO_2_ Coating on Preparation of b-Oriented ZSM-5 Zeolite Film

#### Temperature of Calcination

**Figure 6:**
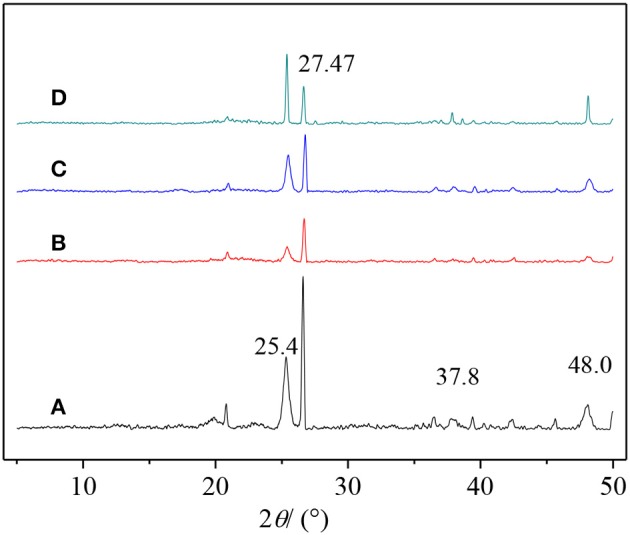
XRD pattern of TiO_2_ coating roast under **(A)** 100°C, **(B)** 300°C, **(C)** 600°C, and **(D)** 900°C.

**Figure 7:**
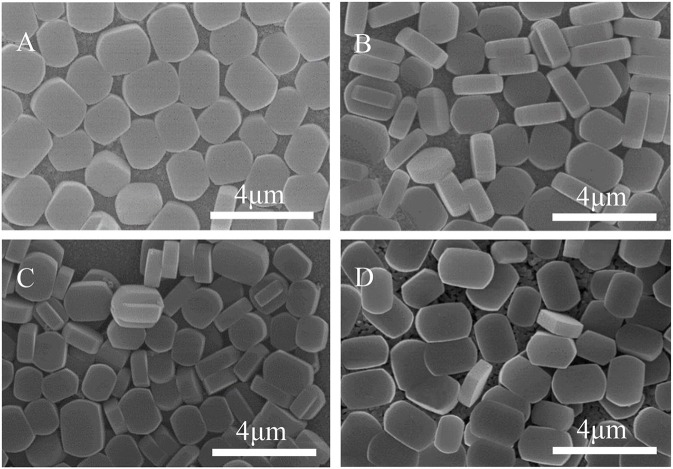
SEM images of ZSM-5 zeolite film on TiO_2_ coating calcine under **(A)** 100°C, **(B)** 300°C, **(C)** 600°C, and **(D)** 900°C.

The Ti-OH species on the substrate surface can be altered by calcining the TiO_2_/substrate. Figure 6 shows the XRD results, pure anatase peaks appear at 2θ = 25.4, 37.8, and 48.0° at 100°C, and the characteristic peak intensity increases with temperature. At 900°C, the diffraction peak is sharp and symmetrical, and the half-width β is narrowed. The crystal form of titanium oxide changes from anatase to rutile with increasing temperature (Liu et al., 2012; Mashimo et al., 2017). Anatase and rutile Ti-OH are more hydrophilic than Ti-OH in mixed crystalline form. The seeds of anatase and rutile TiO_2_ with strong hydrophilicity grow along high b-orientation, and the distribution of crystals on anatase TiO_2_ is very uniform (Figures 7A,B). The thermal treatment is capable of changing the crystal structure of TiO_2_, resulting in various Ti-OH species and different water molecules adsorption mechanisms. The mixed crystal phase (including anatase- and rutile-TiO_2_) is low hydrophilic, and when the temperature rises to 300°C, the intermolecular adsorption mode is converted from non-dissociative adsorption to dissociative adsorption. The TiO_2_ intermolecular force is reduced and partially dissociated, causing a relatively sparse density of TiO_2_ coating on the surface of the substrate. Eventually, the amount of anatase-type Ti-OH is reduced, resulting in partial nucleation to grow along non-b-orientation (Figures 7C,D).

#### Time of UV Irradiation

**Figure 8:**
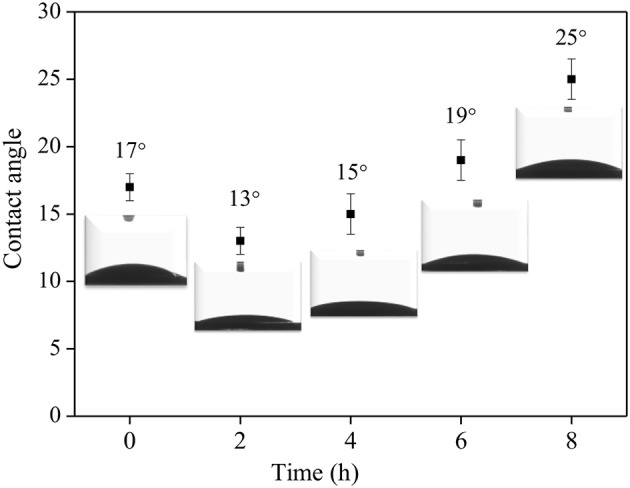
The relationship of UV irradiation time and the contact angles of synthesis solution droplets on TiO_2_ coated substrate.

The effect of surface Ti-OH density on the crystal orientation of ZSM-5 zeolite film is investigated in this section. According to research reports, the hydrophilicity of TiO_2_ films can be tuned by photocatalysis (Mohamad et al., 2015; Barbieriková et al., 2018). In detail, the Ti-OH concentration on the substrate surface was adjusted by irradiating the substrate with ultraviolet light (Wu et al., 2012; Lian and Park, 2016). With the increased UV treatment time, the contact angle of the TiO_2_ coating is gradually reduced, inferring the ultraviolet light irradiation can enhance the hydroxyl concentration on the substrate. Figure 8 shows the contact angle is the smallest when irradiated for 2 h, namely, the hydrophilicity of the substrate is the strongest. TiO_2_ is firstly excited by photons to generate hole-electron pairs (h^+^ and e^−^) (Equation 1), then the bridge oxygen and the positively charged holes (h^+^) react on the surface to cause the breakage of Ti-O bond, and later the bridge oxygens leave the surface to generate oxygen vacancies (Equation 2). At the same time, in order to maintain the surface electrical neutrality, Ti^4+^ is reduced to Ti^3+^(Equation 3), and then the water molecules enter the oxygen vacancy and react to form an adsorbed hydroxyl group (Equation 4). This reaction process can be expressed by the following equations:

(1) $$\begin{array}{r} \text{Photon energy}\to {\text{e}}^{-}+{\text{h}}^{+} \end{array}$$

(2) $$\begin{array}{r} \text{O}{\left(\text{O}\right)}^{2-}+2{\text{h}}^{+}\to \text{V}\left(\text{O}\right)+0.5{\text{O}}_{2} \end{array}$$

(3) $$\begin{array}{r} \text{T}{\text{i}}^{4+}+{\text{e}}^{-}\to \text{T}{\text{i}}^{3+} \end{array}$$

(4) $$\begin{array}{r} {\text{O}}_{\text{neighbor}}+\text{V}\left(\text{O}\right)+{\text{H}}_{2}\text{O}\to 2\text{OH} \end{array}$$

**Figure 9:**
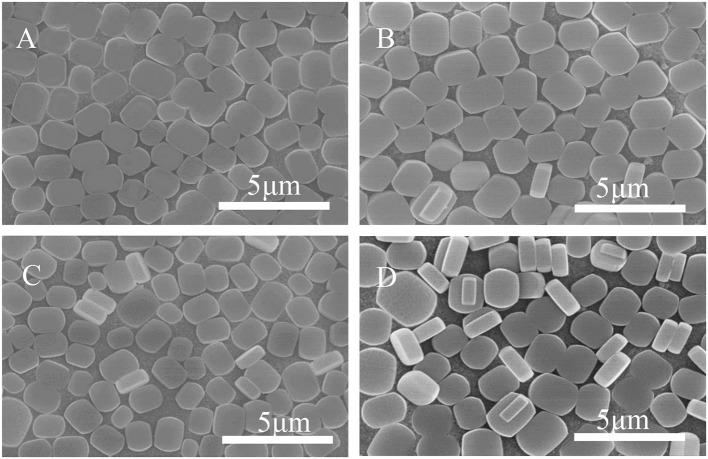
SEM images of ZSM-5 zeolite film on TiO_2_ coated substrate after UV irradiation for **(A)** 2 h, **(B)** 4 h, **(C)** 6 h, and **(D)** 8 h.

Figure 9 shows SEM morphology of the crystal layer synthesized on the TiO_2_ coating irradiated by ultraviolet light at different times. As it can be seen from the figure, the zeolite film is highly uniform with b-orientation on the substrate. When the irradiation time reaches 8 h, a small part of the seed crystal grows on the substrate in the non-b- oriented direction. The extra irradiation results in a decrease in the hydroxyl concentration on the surface of the substrate (Ehhalt and Rohrer, 2000). From Figure 3B, the surface seed crystals without ultraviolet treatment are evenly distributed, but the crystals are not dense enough. In contrast, from Figure 9A, it is found that the crystals on the surface of the substrate are uniformly and densely formed after treated by 2 h ultraviolet irradiation. From Figure 8, it can be seen that under the condition of ultraviolet light irradiation for 2 h, the contact angle is the smallest and the surface hydrophilicity is the strongest, which greatly enhances the Ti-OH concentration and makes the b-oriented ZSM-5 crystal growth denser.

In summary, surface structure and adsorption activity play a crucial role in the orientation and combination of ZSM-5 zeolite film. The introduction of TiO_2_ modification layer provides a great substrate-modification growth interface for ZSM-5 crystal, and enhances the adsorption activity on the substrate and promotes the b-oriented growth of the ZSM-5 zeolite film.

### Textural Properties and Catalytic Performance of ZSM-5/TiO_2_/α-Quartz Zeolite

#### Textural Properties

**Figure 10:**
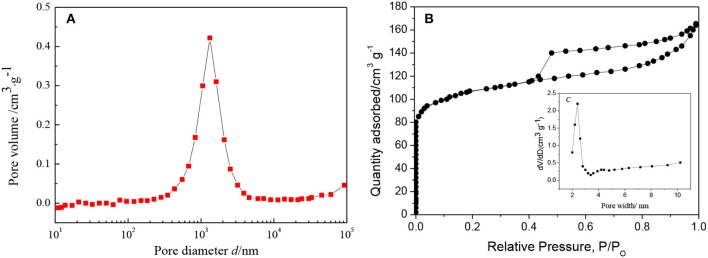
**(A)** Pore size distribution of α-quartz substrate, **(B)** N_2_ adsorption and desorption isotherm for FC zeolite, **(C)** Pore size distribution of ZSM-5 zeolite film.

The change in porosity of the ZSM-5/TiO_2_/α-quartz zeolite (FC) was confirmed by nitrogen adsorption and desorption, Table 2 summarizes the textural properties of the ZSM-5 zeolite before and after loading onto α-quartz substrate, a wide peak (centered around 103 nm) was observed clearly (Figure 10A), The macroporous α-quartz substrate provides a low-resistance mass transfer channel for the raw reactants and products. The FC zeolite exhibits a type IV nitrogen adsorption-desorption isotherm with a low uptake at low relative pressures (Figure 10B), which is consistent with the presence of micropores. And the ZSM-5 pore size distribution agrees with it (Figure 10C). At the same time, there is a typical H4 hysteresis loop in the isotherm at high relative pressure, which is the result of the adsorption and desorption of N_2_ in the mesoporous (Han and Liu, 2015; Chu et al., 2016). During the preparation of FC zeolite, the specific surface area (S_total_) and micropore volume (V_micro_) have different degrees of reduction, but the mesoporous specific surface area (S_meso_) and mesopore volume (V_meso_) increased to 136.6 [m^2^ g^−1^], 0.3455 [m^3^ g^−1^], respectively, this can greatly facilitate the rapid diffusion of the product to the catalyst surface and reduce the formation of coke.

**Table 2:** Specific surface area and pore volume of samples.

	***Area***			***Volume***		
	**Total^**a**^ [m^**2**^ g^**−1**^]**	**Meso^**c**^ [m^**2**^ g^**−1**^]**	**Micro^**b**^ [m^**2**^ g^**−1**^]**	**Total^**a**^ [m^**3**^ g^**−1**^]**	**Meso^**c**^ [m^**3**^ g^**−1**^]**	**Micro^**b**^ [m^**3**^ g−1]**
α-quartz	108.5	–	–	0.6735	–	–
ZSM-5	353.6	73.8	279.8	0.4132	0.0843	0.3289
FC zeolite	325.3	136.6	188.7	0.5802	0.3455	0.2347

a The data are obtained by BET method.

b The data are obtained by t-plot method.

c *The data are the difference between total pore and micropore*.

#### Catalytic Performance

**Figure 11:**
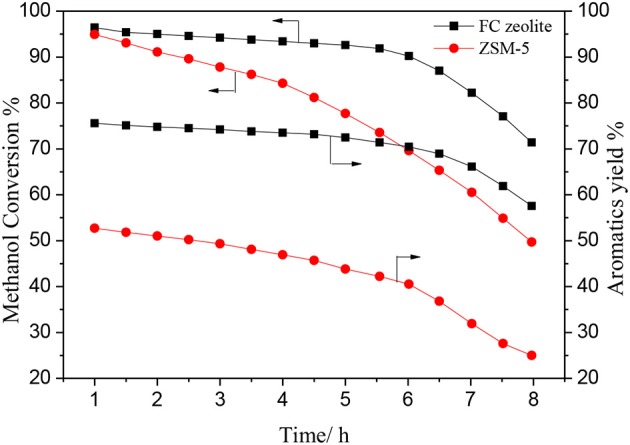
Methanol conversion and aromatics yield of ZSM-5 and FC zeolite in MTA reaction.

Figure 11 shows the methanol conversion and aromatics yield over ZSM-5 and FC zeolite with the reaction time in the MTA reaction. The methanol conversion was similar in the beginning of the reaction, however, after 6 h, the methanol conversion over ZSM-5 was rapidly decreased to 68%, while a slight decrease was observed over b-oriented ZSM-5. Meantime, the aromatics yield over b-oriented ZSM-5 (71%) was much higher than ZSM-5 (40%). The FC zeolite shortens the diffusion path and reduces diffusion resistance for the methanol, alkanes, and aromatic hydrocarbons to rapidly diffuses to the catalyst surface by the mesoporous. However, the single microporous structure has a negative impact on the ZSM-5 catalytic process due to its extremely narrow and elongated micro-channel arrangement which limits mass transfer and facilitated coke formation (Groen et al., 2010; Chu et al., 2019). The introduction of TiO_2_ coating greatly promotes the formation of a dense and uniform b-oriented ZSM-5 zeolite film. The suitable pore structure ensures that the reactants and products in the pore is more conducive to the formation of BTX hydrocarbons, short residence time can reduce the degree of side reaction, thereby maintaining high catalytic activity and selectivity.

## Conclusion

Hydrothermal crystallization method was used to prepare the b-oriented ZSM-5 zeolite film material on the macropore α-quartz substrate. The effects of surface structure and adsorption activity on b-oriented implanting of ZSM-5 nucleus on porous α-quartz substrates were investigated. Simulations of the loading process of ZSM-5 on different modified substrates with TiO_2_, PVA, CTS modifiers were carried out by Forcite module in Material Studio. And we obtained the b-oriented angle and adsorption potential energy though simulations. In the presence of TiO_2_ coating, the ζ-potential energy of the interface of TiO_2_-substrate is zero, forming stable Ti-O-Si bond and strong adsorption potential energy, it turns out to be the most beneficial to ZSM-5 zeolite film loading and growth along the b-orientation.

Moreover, the TiO_2_ coating is subjected to different calcination temperature and ultraviolet irradiation time. With 100°C calcination temperature and 2 h irradiation time, the Ti-OH concentration in the anatase crystal phase reaches the maximum. It is conclusive that the TiO_2_ coating provides a smooth nucleus-substrate growth interface for ZSM-5 zeolite film. Consequently, the interaction between the substrate surface and the ZSM-5 zeolite film is increased and the uniform distribution of the ZSM-5 zeolite film growing along the b-oriented direction is obtained.

Finally, we successfully prepared a FC zeolite with a suitable micro-mesoporous structure. it exhibits excellent catalytic activity and selectivity in MTA reactions and will be valuable material for further application.

## Data Availability Statement

The datasets generated for this study are available on request to the corresponding author.

## Author Contributions

All authors have contributed in various degrees to the analytical methods used, to the research concept, to the experiment design, to the acquisition of data, or analysis, and interpretation of data, to draft the manuscript or to revise it critically for important intellectual content.

### Conflict of Interest

The authors declare that the research was conducted in the absence of any commercial or financial relationships that could be construed as a potential conflict of interest.
